# Cough symptoms in children following COVID-19: a single-center retrospective study

**DOI:** 10.3389/fped.2024.1301571

**Published:** 2024-04-05

**Authors:** Ling Liu, Lu Zhang, Pengxiang Zhou, Wei Zhou, Linghui Li, Lin Zeng, Nan Li, Rongsheng Zhao, Tongyan Han

**Affiliations:** ^1^ Department of Pediatrics, Peking University Third Hospital, Beijing, China; ^2^ Department of Pharmacy, Peking University Third Hospital, Beijing, China; ^3^ Institute for Drug Evaluation, Peking University Health Science Center, Beijing, China; ^4^ Research Center of Clinical Epidemiology, Peking University Third Hospital, Beijing, China

**Keywords:** COVID-19, cough, allergic disease, respiratory infection, children

## Abstract

**Background:**

Cough is the most common respiratory symptom in children with mild coronavirus disease 2019 (COVID-19); however, evidence regarding the duration and severity of COVID-19-related cough is sparse. Herein, we investigated the correlation between cough severity/duration and disease duration in children with allergic diseases following COVID-19.

**Methods:**

This single-center, retrospective case-control study was conducted at the Department of Pediatrics, Peking University Third Hospital, from February 6–13, 2023. Children aged 0–16 completed a questionnaire survey collecting basic information and weekly cough scores for 8 consecutive weeks after COVID-19 in December 2022. The Kaplan–Meier method was used to draw event curves, and the log-rank method was used to compare inter-group differences. Stepwise regression was applied for multivariate analysis of correlations between age, sex, allergic diseases, and the degree and duration of cough following COVID-19.

**Results:**

Overall, 686 children were included, of whom 183 (26.7%) had allergic diseases and 503 (73.3%) did not. Kaplan–Meier analysis identified significant differences between patients with and without allergic disease (log-rank test, *P* = 0.002) and between patients with no allergic disease and those with one and more than one allergic disease (log-rank test, *P* = 0.003). Multivariate regression identified a link between the presence of more than one allergic disease and coughing for >4 weeks after infection (*P* < 0.001). Allergic disease was the primary factor linked to cough symptoms lasting 8 weeks and cough severity (*P* < 0.001).

**Conclusions:**

Allergic disease contributes to the prolonged duration and severity of coughing in children with mild COVID-19.

## 1 Introduction

Since December 2022, Beijing has experienced a rapidly spreading local outbreak of the novel coronavirus disease 2019 (COVID-19). According to the results of genome sequencing analysis, >90% of local infections in Beijing are caused by the omicron variants BA.5.2 and BF.7 (1). According to a preliminary analysis by the Beijing Center for Disease Prevention and Control, 80% of Beijing's permanent residents experienced infection. Although most patients recover quickly, a small number develop persistent symptoms following COVID-19, of which cough is one of the most common symptoms. Patients with allergic diseases such as asthma, allergic rhinitis, and specific dermatitis are more susceptible to airway hyperresponsiveness due to type 2 inflammation. The anaphylactic process by which allergy occurs involves the activation and maturation of dendritic cells in response to exposure to allergens, epithelial alarms, and infectious agents, followed by the clonal expansion of allergen-specific Th2 cells, which are polarized and tend to produce type 2 cytokines (2). However, whether the duration and severity of cough in such patients after COVID-19 are affected by type 2 inflammation and viral infection remains unclear.

In contrast to the manifestations of adult coronavirus, severe infection is rare in children, and mild or asymptomatic infections are more common. In a single-center retrospective study of 171 children with COVID-19, 4% had a cough that did not disappear 3–6 months after infection (3). Another prospective study showed that children with asthma are more likely to develop persistent cough symptoms following COVID-19 (4). At present, most studies on the period after COVID-19 have many limitations, including small sample sizes, lack of a control group, lack of a standardized description of symptoms, lack of detailed stratified analysis, and limited data on children as research participants (5).

To address this knowledge gap, the present study investigated whether allergic diseases could affect the duration and coughing score in children with COVID-19.

## 2 Materials and methods

### 2.1 Study design and participants

This case-control study was designed to explore the cough symptoms, severity, and influencing factors of children with allergic diseases following COVID-19 to allow for optimized targeted treatment and follow-up.

Children aged 0–16 with mild COVID-19 since December 2022 who visited the Department of Pediatrics, Peking University Third Hospital, from February 6–13, 2023, were included in the study. COVID-19 was defined as follows: Clinical manifestations of COVID-19, including dry throat, sore throat, cough, and fever; and presence of one or more of the following etiological and serological test results: (1) Positive COVID-19 nucleic acid test, (2) positive COVID-19 antigen test, (3) positive COVID-19 isolation and culture, and (4) Novel coronavirus specific immunoglobulin G antibody levels four times higher in the convalescent phase than in the acute phase (1). Mild symptoms were defined as the main manifestations of respiratory tract infections, such as dry throat, sore throat, cough, and fever (1).

This study examined the influence of allergic factors on cough in children with COVID-19. The case group comprised children with previous allergic diseases, and the control group comprised children without allergic diseases.

The sample size was calculated using PASS software, using two-sample *t*-tests assuming equal variance and setting the target power to 0.9; we calculated that the study needed to meet the sample size of 46, and the sample size of the case group and the control group were 23 each.

### 2.2 Influencing factors under investigation

The following influencing factors were investigated in the present analysis:

(1) Basic characteristics including sex and age.
(2) Presence of basic diseases: According to the different characteristics of combined allergies, patients were divided into those without allergic diseases and those with allergic diseases (allergic rhinitis, eczema, asthma, allergic conjunctivitis, food allergies, drug allergies). Based on the number of combined allergic diseases, the patients were divided into no allergic disease, one allergic disease, and more than two allergic diseases.
(3) Symptoms of acute COVID-19 including fever, cough, sore throat, dyspnea, abdominal pain, diarrhea, and muscle aches.
(4) Score of cough symptoms over 8 consecutive weeks after COVID-19. Cough scores were assigned to each patient once a week for 8 consecutive weeks after infection to assess the level of cough, as follows: 0, no cough; 1, occasional cough (excluding cough after exercise); 2, frequent cough mildly affecting life or sleep, or cough after exercise; 3, frequent cough seriously affecting life or sleep. According to the cough score, the severity of the cough was graded as follows: 0 and 1 points were mild, and 2 and 3 points were severe.

### 2.3 Data source

A questionnaire survey was conducted, and written informed consent for participation was obtained before the study. This study was approved by the Ethics Committee of the Peking University Third Hospital (approval number: 2023 No. 007-02).

The questionnaire was designed collaboratively between pediatricians, epidemiologists, and medical ethicists. Parents scanned the QR code on their mobile phones, applied the questionnaire star applet, and filled in the requested information. The questionnaire collected data regarding the following characteristics: 1. Basic characteristics, including sex, age, weight, and height; 2. Underlying diseases (whether patients had comorbid allergic diseases); 3. Symptoms of acute COVID-19, including fever, cough, sore throat, dyspnea, abdominal pain, diarrhea, and muscle aches; 4. Weekly cough score after COVID-19 for 8 consecutive weeks. All information was completed before submission, and the quality control officer conducted quality control of the collected information.

SPSS 23.0 software was used to verify the validity and reliability of the questionnaire. Cronbach's *α* and the Kaiser–Meyer–Olkin (KMO) test were employed.

### 2.4 Bias

This study did not investigate infectious factors other than COVID-19 (such as *Mycoplasma pneumoniae* and adenovirus infection) or allergen results. The COVID-19 outbreak in Beijing in December 2022 was a centralized outbreak with a short duration and a centralized research information collection time. As such, environmental factors, infection, and allergen exposure did not affect the results during the observation period.

This study did not include an analysis of medication factors, and the combination of medications was complex. Many drug combinations are associated with underlying diseases in children. Children with allergic diseases, the use of anti-allergic drugs, and the simultaneous inclusion of allergic disease and medication factors may cause the same influencing factors to be analyzed repeatedly.

In the study design, the cough assessment time was measured in weeks, which may have affected the accuracy of the confidence interval in the survival curve analysis. However, using 1 week as the unit is conducive to reviewing children and parents and can more accurately reflect the degree of coughing.

### 2.5 Statistical analysis

R3.5.3 statistical software (R Foundation 3.5.3, Vienna, Austria) was used for data analysis. Counting data are described by case number and/or percentage, inter-group comparisons are described by *X*^2^ statistics, measurement data are shown as the mean ± standard deviation and inter-group comparisons are described by the *U* test. The *t*-test was performed to analyze continuous variables, and the *X*^2^ test was used to analyze categorical variables. The Kaplan–Meier method was performed to draw event curves, and the log-rank method was used to compare the differences in event curves between groups. Cox regression with stepwise selection was used in the multifactor analysis. A *P*-value of <0.05 indicated statistical significance.

## 3 Results

### 3.1 The validity and reliability of the questionnaires

The results showed that the effective questionnaire was 823, the Alpha coefficient was 0.693, and the KMO was 0.802, indicating that the reliability of the questionnaire was acceptable and the validity was appropriate.

### 3.2 Characteristics of the study participants

**Figure 1:**
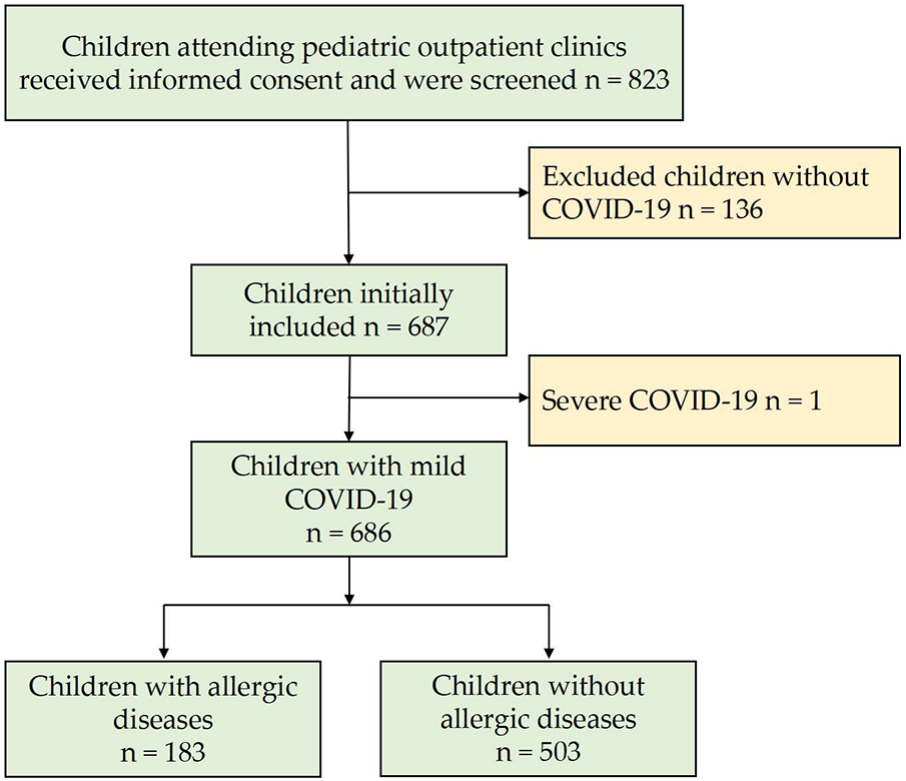
Analysis workflow.

During the study period, 823 children provided informed consent for participation, excluding one case of severe illness and 136 children without novel coronavirus pneumonia. A total of 686 children were included in the study, of whom 183 (26.7%) had allergic diseases and 503 (72.3%) had no allergic diseases. Both the case group and the control group met the sample size requirements. The workflow of the research analysis is shown in Figure 1.

Between February 6 and 13, 2023, 686 children aged 0–16 with mild COVID-19 in December 2022 were enrolled in the pediatric outpatient clinic of Peking University Third Hospital. Basic data of the study participants are shown in Table 1; 388 (56.6%) were boys, and 298 (43.4%) were girls. A total of 183 patients (26.7%) had allergic diseases, while the remaining 503 patients (72.3%) had no allergic disease. Allergic diseases were associated with allergic rhinitis (73.56%), atopic dermatitis (36.54%), asthma (23.56%), allergic conjunctivitis (15.38%), food allergies (37.98%), drug allergies (2.88%), and other allergic diseases (7.21%). Table 2 shows the grouping according to the duration of the cough and influencing factors after COVID-19. Among the children with a cough lasting for more than 4 weeks following COVID-19, 43 (42.2%) were girls, with a median age of 6.75 (0.00–15.9). Allergic diseases were present in 41 cases (40.2%), of which 32 (31.4%) suffered from allergic rhinitis, 12 (11.8%) from asthma, 8 (7.8%) from allergic conjunctivitis, and 20 (19.6%) had atopic dermatitis, 15 (14.7%) had food allergies, 1 (1.0%) had drug allergies, and 3 (2.9%) suffered from other allergic diseases.

**Table 1:** Characteristics of the study participants.

Characteristic	Children (*n* = 686)
Sex	Male 388 (56.6%), Female 298 (43.4%)
Age (median, years)	6.35 (0.01–16)
Presence of allergic diseases	183 (26.7%)
Manifestations of COVID-19	
Fever	643 (93.7%)
Cough	425 (61.95%)
Runny nose	175 (25.52%)
Tachypnea	14 (2.07%)
Chest tightness or shortness of breath	16 (2.31%)
Decreased exercise tolerance	58 (8.51%)

COVID-19, coronavirus disease 2019.

**Table 2:** Grouping and influencing factors of cough duration following COVID-19.

	Cough course >4 weeks	Cough course ≤4 weeks
Sex	Female 43 (42.2%)	Female 255 (43.7%)
Age (median, years)	6.75 (0.00–15.9)	6.25 (0.00–17.8)
Presence of allergic diseases	41 (40.2%)	142 (24.3%)
Allergic pathologies		
Allergic rhinitis	32 (31.4%)	102 (17.5%)
Asthma	12 (11.8%)	36 (6.2%)
Allergic conjunctivitis	8 (7.8%)	19 (3.3%)
Atopic dermatitis	20 (19.6%)	47 (8.0%)
Food allergies	15 (14.7%)	55 (9.4%)
Drug allergies	1 (1.0%)	4 (0.7%)
Other allergic diseases	3 (2.9%)	5 (0.9%)

COVID-19, coronavirus disease 2019.

### 3.3 Kaplan–Meier event curves

**Figure 2:**
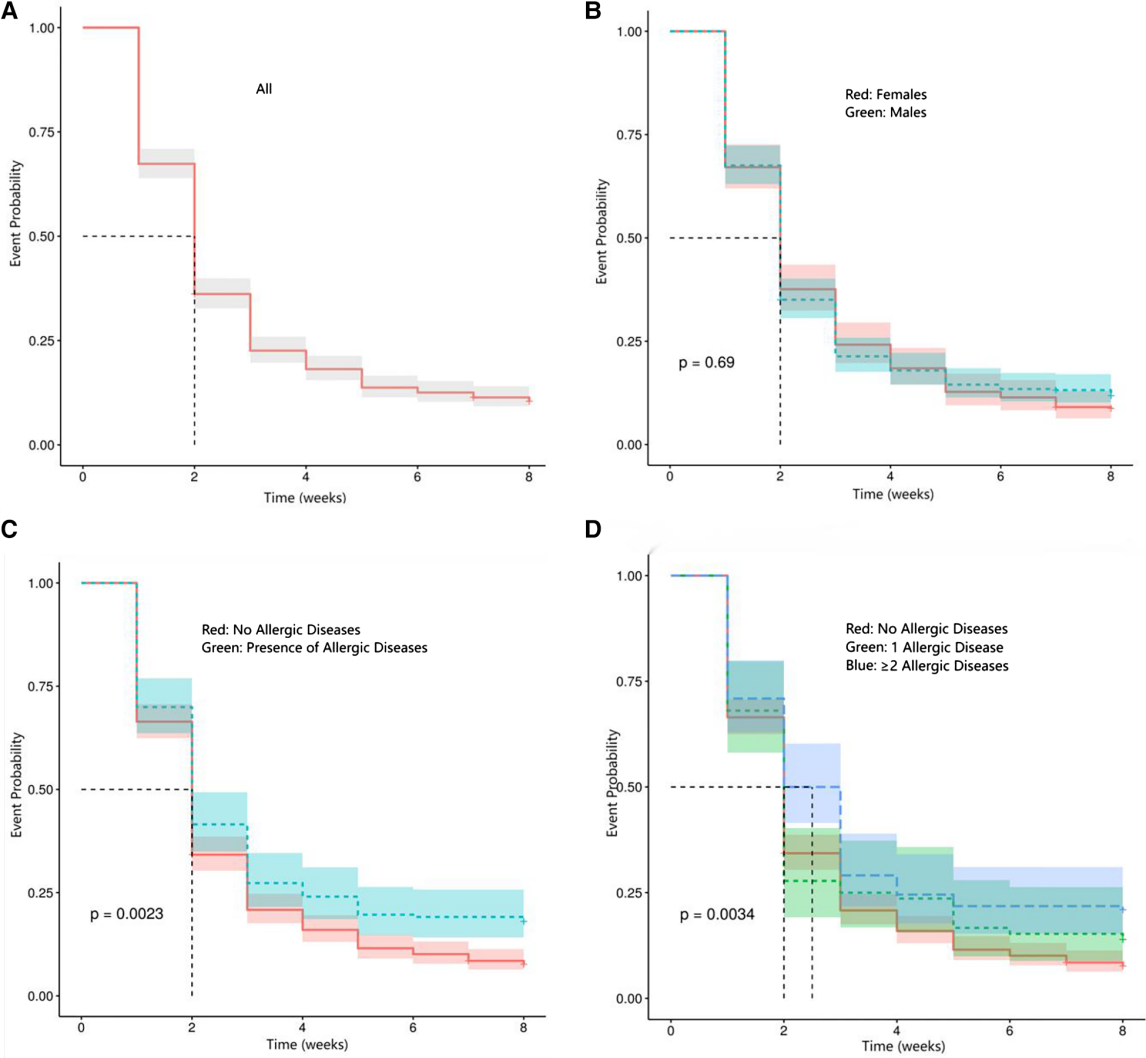
Kaplan–Meier event analyses for cough following COVID-19. (**A**) Cough following COVID-19. (**B**) Cough after COVID-19 across sexes. (**C**) Cough after COVID-19 across groups with and without allergic diseases. (**D**) Cough after COVID-19 in groups stratifies by number of allergic diseases.

Overall, in the 8 consecutive weeks after COVID-19, cough generally showed a downward trend (Figure 2A). The Kaplan–Meier curves did not differ between sexes (Figure 2B). However, differences were found when patients were stratified by the presence and number of allergic diseases (log-rank test, *P* = 0.002 and 0.003, respectively), as shown in Figures 2C, 2D. Kaplan–Meier event analysis showed that the overall non-event rate was 10.45%, *β* = 2 (95% confidence interval, 1.81–2.93), while the mean time to event was 2.82 weeks, with a standard error of 0.09.

### 3.4 Cox regression

*T*-tests were used to analyze continuous variables, and the chi-squared test was used to analyze categorical variables. The *U*-test was used to analyze age as this data did not conform to normality. Age [hazard ratio = 0.98 (0.96, 1.00), *P* = 0.037], presence of allergic diseases [hazard ratio = 0.79 (0.66, 0.96), *P* = 0.015], and number of allergic diseases [hazard ratio = 0.73 (0.58, 0.92), *P* = 0.008] were all closely correlated with symptom prognosis in the 8 weeks following COVID-19 (*P* < 0.05), as shown in Table 3. However, sex [hazard ratio = 0.97 (0.83,1.14), *P* = 0.75] was not correlated with symptom prognosis in the 8 weeks following COVID-19.

**Table 3:** Correlation between factors and multifactor analysis results for cough presence following COVID-19 .

Characteristic	Cough did not disappear (*n* = 74)	Cough disappeared (*n* = 612)	Statistics	*P*	Univariate analysis		Multivariate analysis	
					Hazard Ratio	*P*	Hazard Ratio	*P*
					(95% CI)		(95% CI)	
Age (year)					0.98 [0.96, 1.00]	0.037	0.98 [0.96, 1.00]	0.056
Mean (SD)	7.53 (4.47)	6.37 (4.22)	*U* = 25,888.500	0.0439				
Median [min, max]	7.60 [0.200, 15.9]	6.30 [0.00, 17.8]						
Sex					0.97 [0.83, 1.14]	0.75		
Female	26 (35.1%)	272 (44.4%)	*X*^2 ^= 2.329	0.127				
Male	48 (64.9%)	340 (55.6%)						
Allergic disease					0.79 [0.66, 0.96]	0.015	0.80 [0.67, 0.97]	0.021
None	41 (55.4%)	462 (75.5%)	*X*^2 ^= 13.615	<0.001				
Present	33 (44.6%)	150 (24.5%)						
Number of allergic diseases								
0	41 (55.4%)	463 (75.7%)	*X*^2 ^= 16.115	<0.001				
1	10 (13.5%)	62 (10.1%)			0.91 [0.70, 1.18]	0.468		
≥2	23 (31.1%)	87 (14.2%)			0.73 [0.58, 0.92]	0.008		

COVID-19, coronavirus disease 2019; CI, confidence interval; SD, standard deviation.

Multifactor analysis showed that the presence of allergic diseases was an independent prognostic factor affecting cough in children 8 weeks after COVID-19 (hazard ratio = 0.80, *P* = 0.021), as shown in Table 3. However, age did not affect cough in children 8 weeks after COVID-19 [0.98 (0.96,1.00), *P* = 0.056].

### 3.5 Analysis of risk factors for developing a chronic cough

We analyzed age, sex, the presence and number of allergic diseases, and allergic pathologies as influencing factors. The results of the univariate analysis showed that a history of allergic diseases [hazard ratio = 0.81 (0.67,0.98), *P* = 0.027], the presence of ≥2 allergic diseases [hazard ratio = 0.73 (0.58,0.93), *P* = 0.011], allergic rhinitis [hazard ratio = 0.79 (0.64,0.98), *P* = 0.03], and atopic dermatitis [hazard ratio = 0.70 (0.52,0.95), *P* = 0.021], on the duration of cough >4 weeks had significant differences (all *P* < 0.05, Table 4).

**Table 4:** Cough duration > 4 weeks of overall risk factor analysis results.

Characteristic	Cough course ≤4 weeks (*n* = 584)	Cough course >4 weeks (*n* = 102)	*P*-value
Age (y)			
Mean (SD)	6.40 (4.24)	7.05 (4.33)	0.185
Median [min, max]	6.25 [0.00, 17.8]	6.75 [0.00, 15.9]	
Sex			
Female	255 (43.7%)	43 (42.2%)	0.777
Male	329 (56.3%)	59 (57.8%)	
Allergic disease			
None	442 (75.7%)	61 (59.8%)	<0.001
Present	142 (24.3%)	41 (40.2%)	
Number of allergic diseases			
0	443 (75.9%)	61 (59.8%)	<0.001
1	60 (10.3%)	12 (11.8%)	
≥2	81 (13.9%)	29 (28.4%)	
Allergic pathologies			
Allergic rhinitis	32 (31.4%)	102 (17.5%)	0.03
Asthma	12 (11.8%)	36 (6.2%)	0.376
Allergic conjunctivitis	8 (7.8%)	19 (3.3%)	0.068
Atopic dermatitis	20 (19.6%)	47 (8.0%)	0.021
Food allergies	15 (14.7%)	55 (9.4%)	0.19
Drug allergies	1 (1.0%)	4 (0.7%)	0.896
Other allergic diseases	3 (2.9%)	5 (0.9%)	0.181

SD, standard deviation.

The results of the multivariate analysis showed that the presence of ≥2 allergic diseases was an independent predictor of coughs lasting >4 weeks after COVID-19 [*β* = 0.956, 95% confidence interval (1.575, 4.293), *P* < 0.001]. The allergic pathologies had no effect on coughs lasting more than >4 weeks.

### 3.6 Analysis of risk factors for severe cough lasting for 8 weeks after COVID-19

Analysis of risk factors for developing chronic cough revealed a statistically significant effect between the presence/number of allergic diseases with chronic cough lasting for 8 weeks, and persistent cough severity (all *P* < 0.05, Table 5). However, neither age or sex had a significant effect on coughs lasting for 8 weeks.

**Table 5:** Overall risk factor analysis for risk factors potentially influencing severe cough lasting for 8 weeks following COVID-19.

Characteristic	Mild	Severe	*P*
	(*n* = 668)	(*n* = 18)	
Age (year)			
Mean (SD)	6.49 (4.27)	6.84 (4.03)	0.712
Median [min, max]	6.40 [0.00, 17.8]	5.75 [0.900, 15.7]	
Sex			
Female	293 (43.9%)	5 (27.8%)	0.174
Male	375 (56.1%)	13 (72.2%)	
Allergic disease			
None	495 (74.1%)	8 (44.4%)	0.0112
Present	173 (25.9%)	10 (55.6%)	
Number of allergic diseases			
0	496 (74.3%)	8 (44.4%)	0.011
1	69 (10.3%)	3 (16.7%)	
≥2	103 (15.4%)	7 (38.9%)	

COVID-19, coronavirus disease 2019; SD, standard deviation.

The results of the multivariate analysis showed that the combination of allergic disease was an independent predictor of the duration of cough up to 8 weeks, as well as the severity of cough [*β*=1.27, 95% confidence interval (1.39, 9.21), *P* = 0.008].

## 4 Discussion

COVID-19 has greatly impacted global health, human health, and life, with children also affected by this novel disease. Although the overall clinical manifestations in children are generally mild, and prognoses are good (2), some children develop chronic symptoms after infection, among which respiratory symptoms and cough are the most common (6).

The National Institute for Health and Clinical Excellence guidelines define long COVID-19 as symptoms lasting >4–12 weeks after viral infection, not caused by subsequent infection with other diseases (7). In previous studies, cough was identified as one of the most common symptoms of COVID-19, and many children presented to the hospital within 12 weeks of infection, especially when the cough lasted >4 weeks and had developed into a chronic cough. These cases were often serious enough to attract the attention of pediatricians and require treatment. However, only a few studies have investigated the factors influencing cough in this cohort (8, 9).

In our study, we found that the course and severity of cough in children with mild COVID-19 correlated with the presence of comorbid allergic diseases. Several existing studies on COVID-19 have investigated whether allergy plays a protective role in the acute phase of infection (10–12); however, specific clinical studies on COVID-19 in children are insufficient. This study, therefore, provides valuable clinical evidence of the influencing factors in children with cough caused by COVID-19.

Viral infections, including COVID-19, are also closely associated with autoimmunity, as they can increase airway reactivity and trigger symptoms such as coughing. Allergic diseases in children are characterized by type 2 inflammation, which promotes airway inflammation. Previous studies have elucidated the mechanisms underlying the association between COVID-19 and allergic diseases, showing that COVID-19 causes respiratory symptoms by damaging the airway mucosa and inducing airway inflammation and hypersecretion of airway mucus (13). During infection, viral spikes are inserted into respiratory epithelial cells by binding to the ACE2 receptor, allowing entry into the cells and triggering infection in children. This process requires the promotion of cell entry through proteolytic cleavage involving the proteases transmembrane protease serine 2 (TMPRSS2) and cathepsin L (14, 15). TMPRSS2 reduces the recognition of viruses through human defense mechanisms (16); however, type 2 inflammation, in which interleukin (IL)-4, IL-5, and IL-3 are important inflammatory factors, acts as the inflammatory basis of allergic diseases in children (17). Studies have shown that IL-13 also acts on TMPRSS2, increasing airway mucus production and airway reactivity (14). The present study showed that allergic diseases affect the duration and severity of coughing in children with COVID-19. In children with allergic diseases, cough symptoms persist after infection, sometimes developing into a chronic cough. The results of this study are consistent with the role of inflammation in the aforementioned pathogenesis.

This study has some limitations. First, a sampling survey was conducted. Although information was continuously included during the study period, bias caused by sampling cannot be excluded, and the results may not be universally applicable beyond the study population. Further, the questionnaire was not validated in a larger pilot study. Second, the data relied on parental or self-reported symptoms, which may have introduced a risk of recall bias. However, COVID-19 received widespread attention during this transmission cycle. As a result, the participants were likely to be knowledgeable about symptoms during infection; therefore, recall bias was considered minimal. Third, drug use factors were not analyzed in this study. Drug combinations are complicated, and many drugs are related to underlying diseases in children. Children with allergic diseases use anti-allergy drugs; therefore, allergy and drug-use factors were included simultaneously, and the same influencing factors may be analyzed repeatedly. Fourth, this study did not include infectious factors other than COVID-19 (such as *Mycoplasma pneumoniae* and adenovirus infections) and did not investigate allergen results. Considering that the December 2022 COVID-19 pandemic wave in Beijing was a concentrated outbreak, the epidemic time was short, and the research information collection time was limited. During the study observation period, environmental factors, infection effects, and allergen exposure effects did not cause bias in the results of the observed participants. The results of another drug use analysis on this subject showed that the drug use of the target population during COVID-19 did not affect the cough score. Therefore, factors not included in drug use did not affect the results of this study.

In addition to type 2 inflammation, other inflammatory pathways between COVID-19 and allergic diseases warrant further exploration. Further, for children with COVID-19, the problems of airway remodeling and changes in airway structure after infection also deserve more attention, in addition to the association between viral infection and the immune system.

In conclusion, allergic diseases may be associated with the subsequent course and severity of coughing in children with COVID-19. However, these results need to be confirmed in future studies with larger sample sizes. Clinicians should consider that persistent coughing may be prolonged following COVID-19 in children with allergies. Children with severe cough should be differentiated from those with allergic diseases and administered symptomatic medication.

## Data Availability

The raw data supporting the conclusions of this article will be made available by the authors, without undue reservation.
